# Differential privacy protection algorithm for network sensitive information based on singular value decomposition

**DOI:** 10.1038/s41598-023-33030-4

**Published:** 2023-04-13

**Authors:** Xuan Ma, Xing Chang, Hongxiu Chen

**Affiliations:** 1 College of Physics Science and Technology, Shenyang Normal University, Shenyang, 110034 Liaoning China; 2 Shenyang Instiute of Computing Technology Co.Ltd.CAS, Shenyang, 110168 Liaoning China

**Keywords:** Engineering, Mathematics and computing

## Abstract

In order to reduce the risk of data privacy disclosure and improve the effect of information privacy protection, a differential privacy protection algorithm for network sensitive information based on singular value decomposition is proposed. TF-IDF method is used to extract network sensitive information text. By comparing the word frequency of network sensitive information, high word frequency word elements in network information content are collected to obtain the mining results of network sensitive information text. According to the decision tree theory, the equal difference privacy budget allocation mechanism is improved to achieve equal difference privacy budget allocation. By discarding some small singular values and corresponding spectral vectors, the data can be disturbed, and the availability of the original data can be retained, so that it can truly represent the original data set structure. According to the results of equal difference privacy budget allocation and singular value decomposition disturbance, the data of high-dimensional network graph is reduced by random projection, singular value decomposition is performed on the reduced data, and Gaussian noise is added to the singular value. Finally, the matrix to be published is generated through the inverse operation of singular value decomposition to achieve differential privacy protection of network sensitive information. The experimental results show that the privacy protection quality of this algorithm is high and the data availability is effectively improved.

## Introduction

In the context of the rapid development of Internet technology, data shows an explosive growth trend and is flooding in various fields. According to statistics, the daily average number of microblog releases exceeds 270 million, and the daily average number of searches by Baidu search engine exceeds 1.54 billion. Various social software, online shopping platforms, management systems, etc. are also producing data closely related to people's lives. This data include personal consumption records, travel records, medical records, etc. There is no doubt that these data are very important for data analysts. Especially with the help of diversified data mining^1,2^ and other technologies, data analysts can mine more valuable data. For example, through statistical analysis of the records of users' purchase of health care products, it is possible to find out "the age stratification of purchase of health care drugs" or the demand for health care drugs. However, this data contains a large amount of personal private information. Once released directly, it will directly lead to the disclosure of users' personal privacy information, which will not only bring users property information security losses, but also bring legal and economic disputes to the data publisher; On the other hand, even after encryption, privacy protection and other technical processing, attackers can still use data mining, machine learning and other technologies to steal users' personal privacy information. Therefore, the problem of privacy protection needs to be solved urgently^3,4^.

Under the above background, literature^5^ proposed a differential privacy protection method for sensitive information of the population intelligent perception network. Considering the coupling relationship between the perceived information availability and privacy protection, the perceived information quality and privacy protection are optimized respectively. First, calculate the dispersion rule of the noise range, use Bayes to derive the best estimate of noise dispersion, adjust the noise range of information perception, and search for the best estimated information probability matrix through iterative processing to improve the quality of perceived information. Then, optimize the differential privacy, introduce noise mechanism and sensitivity constraints, and eliminate the correlation between the differential privacy algorithm and the information set. Finally, considering the difference of information types in perceptual information, design corresponding processing mechanisms for numerical and non numerical information respectively. The simulation results show that this method can effectively improve the availability of perceptual information, and has better privacy processing accuracy and execution efficiency. Literature^6^ proposed a personalized differential privacy protection method based on Skyline computing. First, the attribute vector of users was constructed, and then the method based on Skyline computing was used to evaluate the privacy disclosure level of users, and the user data set was segmented according to this level. Then, the sampling mechanism is applied to achieve personalized differential privacy, and noise is added to the integrated data. Finally, the security and practicality of the processed data are analyzed and published. The experimental results verify that this method has a high quality of privacy protection and has certain application value. Literature^7^ proposed a privacy protection method for sensitive data based on migration learning, which combines multiple "master" models trained from disjoint sensitive data sets in a "black box" way, and these models directly depend on sensitive training data. The "apprentice" is obtained from the "master" collection transfer learning, and cannot directly access the "master" or basic parameters. The data domain of the "apprentice" is different from but related to the sensitive training data domain. In terms of differential privacy, attackers can query "apprentices" or check their internal work, but they cannot obtain the privacy information of training data. Literature^8^ proposes a virtual text retrieval privacy protection method. Use carefully designed pseudo queries to mask user queries, thereby protecting user privacy. Designing a system framework based on the client side to protect user privacy does not require changing existing text retrieval algorithms or compromising the accuracy of text retrieval. Define user privacy models to establish the requirements that ideal pseudo queries should meet to achieve sensitive information protection. Literature^9^ proposes an effective cloud based security management solution, with the basic idea of deploying a trusted local server between an untrusted cloud and each trusted client of a medical information management system, responsible for running the electronic medical record cloud hierarchical storage model and the electronic medical record cloud segmentation query model. The electronic medical record cloud hierarchical storage model is responsible for storing lighter electronic medical record data items on local servers, while encrypting and storing highly private electronic medical records on the cloud to ensure the confidentiality of patient privacy information in the electronic medical records on the cloud. Literature^10^ proposes a basic framework for comprehensively protecting the privacy of various users in personalized information retrieval. Its basic idea is to construct a set of carefully designed fake requests, and submit them to the server together with each user request to confuse user requests, and then mask the user privacy behind the requests, achieving security protection for various types of user privacy information.

Although the above methods play a role in privacy protection to a certain extent, there are still problems of poor privacy protection quality and data availability in practical applications. Therefore, this paper proposes a differential privacy protection algorithm for network sensitive information based on singular value decomposition. The organization process of this article's research content is as follows: Firstly, TF-IDF method is used to extract network sensitive information text, collect word elements with high word frequency, and obtain the mining results of network sensitive information text. Secondly, based on the decision tree theory, the equal difference privacy budget allocation mechanism is improved to achieve equal difference privacy budget allocation, which can not only preserve the availability of the original data, but also truly reproduce the structure of the original dataset. Random projection is used to perform dimensionality reduction and singular value decomposition on high-dimensional network graph data, and inverse operations are used to generate a matrix to be published, achieving differential privacy protection for sensitive network information. The test results prove that the contribution of the algorithm designed in this paper lies in improving the quality of privacy protection and effectively improving the availability of network data.

## Network sensitive information mining

### Access to network sensitive information text

The TF-IDF method is used to extract network sensitive information text. By comparing the word frequency of network sensitive information, the high word frequency word elements in network information content are collected, and then the network sensitive information text is obtained. The main idea of TF-IDF method is that if a word or phrase is generated frequently in one article and rarely appears in other articles, it indicates that the word or phrase has good classification ability and can be used for classification. TF-IDF includes TF (Term Frequency) and IDF (Inverse Document Frequency). TF indicates the frequency of sensitive words in document $d$. The main idea of IDF is that if there are fewer documents containing sensitive word $r$ and the IDF is larger, then sensitive words have good classification ability. The process of using TF-IDF method to obtain network sensitive information text is shown in formula (1):

1 $$ w_{dr} = TF_{dr} + IDF_{dr} $$

In formula (1), $w_{dr}$ represents the proportion of sensitive word $r$ in document $d$, that is, the obtained network sensitive information text; $TF_{di}$ represents the frequency of word $i$ in document $d$; $IDF_{dr}$ represents the frequency of sensitive word $r$ in document $d$. The expressions of $TF_{di}$ and $IDF_{dr}$ are shown in formula (2) and formula (3):

2 $$ TF_{di} = \frac{{F_{di} }}{{\max \left\{ {F_{hi} |h = 1,2,...,T} \right\}}} $$

3 $$ IDF_{dr} = \sqrt {\frac{N}{{n_{r} }}} $$

In formula (2) and formula (3), $F_{hi}$ represents the document $d$ contains $T$ keywords; $N$ represents the total number of documents; $n_{r}$ represents the total number of documents containing the sensitive word $r$.

After obtaining the network information text through the TF-IDF method based on the above analysis, the representative sensitive information features should be selected. Use formula (4) to calculate the characteristics of sensitive information:

4 $$ f_{d} = \frac{{\sum\limits_{i,r = 1}^{N} {TF_{di} + IDF_{dr} } }}{{W_{dr} \times p_{r} \times M_{k} }} $$

In the formula (4), $W_{dr}$ represents network sensitive information text; $p_{r}$ represents the proportion of sensitive information in all network information; $M_{k}$ represents the number of sensitive information features in all network information; $k$ represents the quantity of sensitive information; $M$ represents the quantity of all network information.

### Realize the mining of network sensitive information

The network sensitive information feature $f_{d}$ obtained by the above method can be described by $F = \left\{ {f_{d1} ,f_{d2} ,...,f_{dl} } \right\}$. In this set, $d = 1,2,...,D$, $l = 1,2,...,k$. Select a sensitive information feature $F$ randomly from the above set, and its corresponding characteristic value is shown in formula (5):

5 $$ F^{2} = \left[ {\begin{array}{*{20}c} {f_{11} } & {f_{12} } & {...} & {f_{1l} } \\ {f_{21} } & {f_{22} } & {...} & {f_{2l} } \\ \vdots & \vdots & \ddots & \vdots \\ {f_{k1} } & {f_{k2} } & {...} & {f_{kl} } \\ \end{array} } \right] $$

In the above network sensitive information features, $c$ samples are collected and set as the cluster center. When mining network sensitive information, a reasonable threshold should be set to complete the mining of network sensitive information based on the clustering results. The detailed process is as follows:

Set $A_{js} \left( {j = 1,2,...,c;s = 1,2,...,r} \right)$ to represent the $s$ characteristic parameter of the $j$ cluster center of sensitive information in the network. The characteristic membership of sensitive information is obtained by formula (6):

6 $$ A_{js} \left( n \right) = Q^{2} \times \frac{{\phi_{j}^{s} }}{{F^{2} }} $$

In the formula (6), $Q^{2}$ represents the upper limit of network information quantity; $\phi_{j}^{s}$ represents the similarity between sensitive information of different attributes in the network.

The Euclidean distance between sensitive information features is obtained by formula (7) ^11^:

7 $$ dis\left( n \right) = F^{2} \times \sqrt {\sum\limits_{j,s = 1}^{N} {A_{js} } } $$

Obtain the maximum distance between sensitive information and normal information through formula (8):

8 $$ d_{\max } = \sum\limits_{j = 1}^{N} {\sum\limits_{s = 1}^{N} {dis_{js} \left( n \right)} } $$

Then the cluster center of network sensitive information mining is obtained through formula (9) to realize the mining of network sensitive information:

9 $$ G_{r} = d_{\max } \int\limits_{r = 1}^{N} {dis_{ij} \left( n \right)} \times n_{r} $$

Through the above analysis, the mining results of network sensitive information are obtained, and the singular value decomposition method is used for the mining results to achieve the differential privacy protection of network sensitive information.

## Differential privacy protection of network sensitive information

How to protect network sensitive information under the premise of effectively mining network sensitive information is an important topic in the field of data processing. Singular value decomposition (SVD)^12,13^ is a common data mining matrix decomposition method and information retrieval method. Singular value decomposition belongs to a matrix decomposition in linear algebra and is widely used in the field of machine learning to reduce the dimensions of data sets. Singular value decomposition (SVD) can not only eliminate data noise, but also achieve effective privacy protection. Therefore, this paper uses the singular value decomposition method to study the differential privacy protection of network sensitive information.

### Equal privacy budget allocation mechanism

The differential privacy mechanism mainly protects data privacy by adding noise to the dataset. Therefore, the reasonable allocation of the privacy budget is an important point in the algorithm design process. At present, most of the existing methods adopt the traditional privacy budget allocation scheme, that is, half of the remaining privacy budget is allocated to each decision node and leaf node layer by layer. At this time, the privacy budget and added noise of each layer of the differential privacy protection algorithm are the same. With the continuous increase of the number of decision tree layers, the privacy budget allocated to each layer of decision nodes decreases exponentially, so the noise of adding query results becomes larger. At the same time, because the number of sample datasets included in the leaf node is small, the classification accuracy of this node will decline, although the privacy of the data is guaranteed during the operation, the performance of the algorithm is greatly affected and is not applicable to practice. Therefore, this paper improves the existing equal difference privacy budget allocation mechanism according to the decision tree theory^14^ to improve the quality of privacy protection.

Specifically, the equal difference privacy budget allocation mechanism is as follows: set the overall privacy budget of the algorithm as $\eta$, and the recursion number of the algorithm as $\alpha$. When the data is located at the level $x$ of the decision tree, the privacy budget allocated to the current level is shown in formula (10):

10 $$ \eta_{x} = \frac{\eta }{{\alpha \left( {\alpha + 1} \right)}} $$

According to the unique sequential combination characteristic of differential privacy, the privacy protection budget consumed by the algorithm as a whole is equal to the sum of privacy protection budgets consumed by each layer of the decision tree. However, since the data sets used by nodes in each layer for splitting and counting are disjoint, according to the parallel combination characteristics of differential privacy, the privacy budget allocated to each level does not need to be evenly distributed to each node, and each node is directly given the same and equal to its own privacy budget. In the part of the algorithm that adds noise to protect data privacy, because the Laplacian mechanism can better fit the random distribution of noise, the most commonly used Laplacian mechanism is selected to add noise to the count value of data in the leaf node. At the same time, considering that there may be continuous features in the dataset, the algorithm also uses an exponential mechanism to calculate the Gini index of continuous features, so as to select the best continuous features and split points.

In the isochromatic privacy budget allocation, perform the following operations for each decision tree:

(1) Extract the same amount of data set from the training data set;

(2) Repeat the following steps until the stop conditions are met as follows;

1. If the data on the node meets the stop condition, set the node as a leaf node, stop creating sub nodes, use the Laplacian mechanism to add noise to the node's count value, and then select the classification with the most samples as the label of the leaf node;
2. If the data on the node does not meet the stop condition, calculate the number of data sets contained in the node, and use the Laplace mechanism to add noise;
3. Randomly select some features from the feature set. If the sub feature set contains continuous features, execute 4); otherwise, end the algorithm;
4. The privacy protection budget is equally allocated to each feature, and an exponential mechanism is used to select the best splitting feature and mode from all continuous features;
5. Calculate the Gini index of each remaining discrete feature on the data set, and compare it with the Gini index of the local optimal feature to select the global optimal splitting feature and split the nodes.

(3) If the stop condition is met, equal difference privacy budget allocation can be realized.

### Singular value decomposition disturbance

Singular value decomposition (SVD) is not a new technology in the application of differential privacy protection for network sensitive information, but its application in the perturbation of information privacy protection is emerging recently. A remarkable feature of singular value decomposition (SVD) is to reduce the dimension of compressed data while maintaining the main data patterns. The main purpose of matrix decomposition is to obtain some low dimensional, approximately related data structures of objects and attributes from the original dataset.

Let $B$ be any real matrix of order $m \times n$, i.e. $B \in P^{m \times n}$, then there is an orthogonal matrix $Q$ of order $m \times m$, a generalized diagonal matrix $m \times n$ of order $V$ and an orthogonal matrix $G$ of order $n \times n$, so that the $B$ is shown in formula (11):

11 $$ B = \left( {QVG} \right)^{T} $$

The remarkable feature of singular value decomposition is that it protects the main data patterns while reducing the dimension of compressed data. In the differential privacy protection of network sensitive information, the disturbed dataset $B_{h}$ can preserve the availability of the original data while providing data privacy protection, so that it can truly represent the original dataset structure.

Singular value decomposition (SVD) in AI domain is used as a data perturbation strategy to protect data privacy. An undirected weighted network with $M$ nodes and $L$ edges can be expressed as $Y\left( {Z,O} \right)$, where $Z$ represents the set of nodes, $O$ represents the set of edges, the number of nodes $M = \left| Z \right|$, and the number of edges $L = \left| O \right|$. If element $o_{ij}$ exists in edge set $O$, it indicates the edge between node $o_{i}$ and node $o_{j}$. In weighted network $Y$, each edge $o_{ij}$ has a weight $w_{ij}$ corresponding to it. If $Y$ has no multiple edges, $Y$ can be represented by its adjacency matrix $B = P^{n \times n}$. The $B\left( {i,j} \right)$ is shown in formula (12):

12 $$ B\left( {i,j} \right) = b_{ij} = \left\{ \begin{gathered} 0,{\kern 1pt} {\kern 1pt} {\kern 1pt} {\kern 1pt} {\kern 1pt} {\kern 1pt} {\kern 1pt} {\kern 1pt} {\kern 1pt} {\kern 1pt} {\kern 1pt} {\kern 1pt} {\kern 1pt} o_{ij} \notin O \hfill \\ w_{ij} ,{\kern 1pt} {\kern 1pt} {\kern 1pt} {\kern 1pt} {\kern 1pt} o_{ij} \in O \hfill \\ \end{gathered} \right. $$

For the adjacency matrix $B = P^{n \times n}$ of a weighted network $Y$, there must be a complete singular value decomposition:

13 $$ B \in P^{n \times n} $$

SVD disturbance mainly perturbs the data by discarding some of its smaller singular values and corresponding spectral vectors. If diagonal matrix $\omega_{j}$ is diagonal matrix after discarding $\delta$ ($\delta \ge 0$) smaller singular values, the $\omega_{j}$ is shown in formula (14):

14 $$ \omega_{j} = diag\left[ {\omega_{1} ,\omega_{2} ,...,\omega_{N - j} ,...,0} \right] $$

Then the adjacency matrix after SVD disturbance is shown in formula (15):

15 $$ B_{j} = \left( {Q\omega_{j} G} \right)^{T} $$

In order to strengthen the protection of data, $Q$ and $G$ matrices are further processed based on SVD disturbance, and this method of double disturbance of data is called sparse singular value decomposition. The specific process of SSVD is as follows:

Disturb the singular value of adjacency matrix $B$ to get $B_{j}$, and then let:

16 $$ \overline{Q} \left( {i,j} \right) = \left\{ \begin{gathered} 0,{\kern 1pt} {\kern 1pt} {\kern 1pt} {\kern 1pt} {\kern 1pt} {\kern 1pt} {\kern 1pt} {\kern 1pt} {\kern 1pt} {\kern 1pt} {\kern 1pt} {\kern 1pt} {\kern 1pt} {\kern 1pt} {\kern 1pt} {\kern 1pt} {\kern 1pt} {\kern 1pt} {\kern 1pt} {\kern 1pt} {\kern 1pt} {\kern 1pt} {\kern 1pt} {\kern 1pt} {\kern 1pt} {\kern 1pt} {\kern 1pt} {\kern 1pt} {\kern 1pt} {\kern 1pt} {\kern 1pt} {\kern 1pt} {\kern 1pt} {\kern 1pt} {\kern 1pt} {\kern 1pt} \left| {Q\left( {i,j} \right)} \right| < \tau \hfill \\ Q\left( {i,j} \right),{\kern 1pt} {\kern 1pt} {\kern 1pt} {\kern 1pt} {\kern 1pt} {\kern 1pt} {\kern 1pt} {\kern 1pt} \left| {Q\left( {i,j} \right)} \right| \ge \tau \hfill \\ \end{gathered} \right. $$

17 $$ \overline{G} \left( {i,j} \right) = \left\{ \begin{gathered} 0,{\kern 1pt} {\kern 1pt} {\kern 1pt} {\kern 1pt} {\kern 1pt} {\kern 1pt} {\kern 1pt} {\kern 1pt} {\kern 1pt} {\kern 1pt} {\kern 1pt} {\kern 1pt} {\kern 1pt} {\kern 1pt} {\kern 1pt} {\kern 1pt} {\kern 1pt} {\kern 1pt} {\kern 1pt} {\kern 1pt} {\kern 1pt} {\kern 1pt} {\kern 1pt} {\kern 1pt} {\kern 1pt} {\kern 1pt} {\kern 1pt} {\kern 1pt} {\kern 1pt} {\kern 1pt} {\kern 1pt} {\kern 1pt} {\kern 1pt} {\kern 1pt} {\kern 1pt} {\kern 1pt} \left| {G\left( {i,j} \right)} \right| < \tau \hfill \\ G\left( {i,j} \right),{\kern 1pt} {\kern 1pt} {\kern 1pt} {\kern 1pt} {\kern 1pt} {\kern 1pt} {\kern 1pt} {\kern 1pt} \left| {G\left( {i,j} \right)} \right| \ge \tau \hfill \\ \end{gathered} \right. $$

where $\tau$ represents the lower bound of the absolute value of the value in the $Q$ and $G$ matrices. Then the matrix after disturbance is shown in formula (18):

18 $$ \overline{{B_{j} }} = \left( {\overline{Q} \omega_{j} \overline{G} } \right)^{T} $$

$\tau$ can limit the size of elements in the matrix. In different types of networks, the distribution of values of $Q$ and $G$ matrices may vary greatly, which leads to the failure to evaluate the proportion of values discarded in $Q$ and $G$ matrices under the same condition of $\tau$. In order to better adapt to different types of networks and make the number of discarded values in $Q$ and $G$ more controllable, $\vartheta$ in this paper is defined as the proportion of deleted values in $Q$ and $G$, is shown in formula (19):

19 $$ \vartheta = \frac{{N_{\tau } }}{N \times M} $$

In formula (19), $N_{\tau }$ represents the number of discarded values in $Q$ or $G$ matrix.

### Implementation of differential privacy protection for network sensitive information

According to the results of equal difference privacy budget allocation and singular value decomposition perturbation, a differential privacy protection algorithm for network sensitive information based on singular value decomposition is further proposed. In the first step, random projection is used to reduce the dimensions of high-dimensional network graph data. In the second step, singular value decomposition is performed on the reduced dimension data and Gaussian noise is added to the singular value. Finally, the matrix to be published is generated through the inverse operation of singular value decomposition. Compared with adding Gaussian noise directly to the reduced dimension data, adding Gaussian noise to the values in the singular value matrix can effectively reduce the amount of noise added.

Assuming that the attacker has strong background knowledge, the Euclidean distance information of sensitive information in the network will not be disclosed. According to network $Y$ given above, publish a matrix $B$ to be published that meets differential privacy, and retain the structural characteristics of the original network as much as possible to improve the availability of information. If the rank of matrix $B$ is $\sigma$, then there are unitary matrices $Q^{\prime}$ and $G^{\prime}$, that is, left singular matrices and right singular matrices, so that:

20 $$ B = \left[ {Q^{\prime}\left( {\begin{array}{*{20}c} {\omega_{j} } & 0 \\ 0 & 0 \\ \end{array} } \right)G^{\prime}} \right]^{T} $$

Let $\psi$ and $\psi ^{\prime}$ be two matrices with the same singular value number, and:

21 $$ \left\{ \begin{gathered} \psi_{1} \ge \psi_{2} \ge ... \ge \psi_{s} \hfill \\ \psi_{1}^{^{\prime}} \ge \psi_{2}^{^{\prime}} \ge ... \ge \psi_{s}^{^{\prime}} \hfill \\ \end{gathered} \right. $$

Then for any unitary invariant norm $\left\| \cdot \right\|$ is shown in formula (22):

22 $$ \left\| {diag\left( {\psi_{i}^{^{\prime}} - \psi_{i} } \right)} \right\| \le \left\| {\psi ^{\prime} - \psi } \right\| $$

It can be seen from the above analysis that considering the problem that traditional methods will directly add Gaussian noise when reducing high-dimensional data to low dimensional data, resulting in a large amount of noise, this paper proposes a differential privacy protection algorithm for network sensitive information based on singular value decomposition. Reducing the dimensions of the noise matrix can reduce the amount of noise added, so that the expected value after disturbance is lower. Therefore, singular value decomposition is introduced to decompose the projected matrix. Gaussian noise is added to the singular value and less noise is added to improve the data availability.

This method combines random projection^15^ and singular value decomposition to solve the problem of large amount of noise added by traditional methods. First, this method generates the adjacency matrix of social network, uses random projection to convert high-dimensional matrix into low dimensional matrix, then decomposes the low dimensional matrix into left singular matrix, right singular matrix and singular value matrix, and finally adds Gaussian noise to the singular value matrix, the matrix to be published is generated according to the inverse operation of singular value decomposition. The method in this paper adds noise to the singular value matrix, because the singular value matrix only has values on the main diagonal, and the number of values is the rank of the matrix. Compared with the $m \times n$-dimension noise matrix in the traditional method, it effectively reduces the amount of noise when adding data sets.

**Figure 1:**
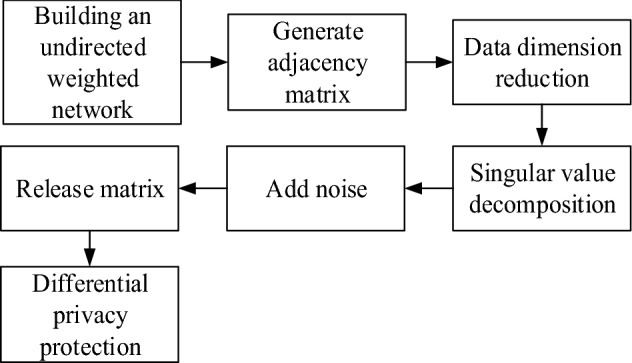
Flow chart of differential privacy protection for network sensitive information.

The implementation process of differential privacy protection for network sensitive information is shown in Fig. 1.

According to Fig. 1, the specific steps for differential privacy protection of network sensitive information are given:

1. Preprocess the original network graph and calculate its adjacency matrix $B$;
2. Generate unitary matrices $Q^{\prime}$ and $G^{\prime}$, and let $$\left\{ \begin{gathered} \psi_{1} \ge \psi_{2} \ge ... \ge \psi_{s} \hfill \\ \psi_{1}^{^{\prime}} \ge \psi_{2}^{^{\prime}} \ge ... \ge \psi_{s}^{^{\prime}} \hfill \\ \end{gathered} \right.$$;
3. Use matrix $Q^{\prime}$ and $G^{\prime}$ to calculate matrix $B^{\prime}$ after projection;
4. Singular value decomposition of matrix $B^{\prime}$;
5. Gaussian noise is added to singular value;
6. Discard some values in $Q$ and $G$, reorder singular values, and obtain a new singular value matrix;
7. The matrix to be published is generated according to the inverse operation of singular value decomposition to achieve the differential privacy protection of network sensitive information.

## Experimental study

### Experimental environment and sample data

In order to verify the effectiveness and feasibility of the differential privacy protection algorithm for sensitive network information based on singular value decomposition, experimental research was carried out. The experimental environment of this paper is macOS 10.13.1 operating system, the development environment is IntelliJ IDEA, the programming language is Java, and the experimental data is analyzed using Matlab simulation software. The algorithm was written using the neural network toolbox in the MATLAB companion toolbox.

A Inspur blade server is used to simulate the deployment of multi tenant applications, configured with 12 core CPU (primary frequency 3.10 GHz), 32 GB memory, and 2 TB hard disk. The storage layer uses MySQL 5.6.22 as the storage database, and multi tenant applications use Java 1.8 to write programs for simulation. The data set is taken from the data in the basic medical registration information table of the insured in the internal test data set of the social security project, and the data volume is about 3 million pieces. The data selected a total of 20 attributes including family name (fuzzed), medical personnel category, gender, age, trust level, widowhood category, company organization, medical account and other attributes for the experiment. Incompatible privacy constraints are family name, gender, age and company organization.

### Experimental results

**Figure 2:**
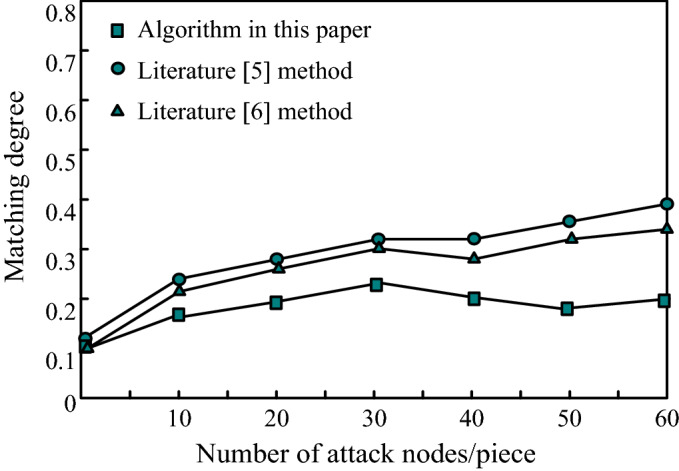
Comparison results of attack results.

The verification method is to take the published graph after privacy protection as a sub graph, and check the matching degree between the published results under the sub graph attack and the real dataset. The lower the matching degree, the better the privacy protection effect. First, it is assumed that the attacker has the background knowledge of the target and incomplete subgraph information; Then, the node identification attack is carried out; Finally, the methods in literature^5^, literature^6^ and the network graph published under the algorithm in this paper are used as subgraphs respectively to compare the attack results under the two kinds of background knowledge. The matching degree of attack results is shown in Fig. 2.

It can be seen from Fig. 2 that under the node attack, the maximum match degree of the attack results of the literature^5^ method reaches 4.1, the maximum match degree of the attack results of the literature^6^ method reaches 3.7, while the maximum match degree of the attack results of the algorithm in this paper is only 2.3. It can be seen from the comparison that the data matching degree of the algorithm proposed in this paper under node attack is obviously smaller than the methods in literature^5^ and literature^6^, indicating that the data availability of the algorithm proposed in this paper is relatively good, that is, the privacy protection quality of the algorithm proposed in this paper is higher.

**Figure 3:**
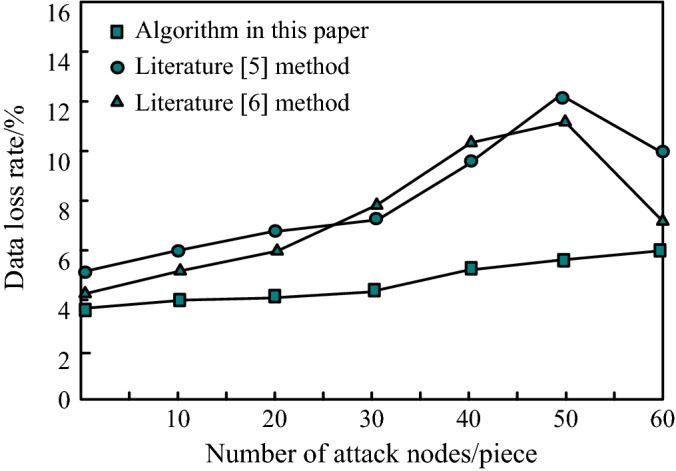
Comparison results of data loss rate.

How to avoid data destruction on the premise of ensuring data privacy is the key to verify the effectiveness of the method. Calculate the data loss rate and evaluate the data availability according to this indicator. The comparison results of data loss rates are shown in Fig. 3.

It can be seen from Fig. 3 that when there are 60 node attacks, the data loss rate of the algorithm in this paper reaches the maximum value, 5.8%; When the number of node attacks is 50, the data loss rate of the method in literature^5^ reaches the maximum, which is 12.5%; When there are 50 node attacks, the data loss rate of the method in literature^6^ reaches the maximum, which is 11.3%. The above experimental results show that the data loss rate of this algorithm is low, indicating that this algorithm can not only improve the quality of data privacy protection, but also reduce data loss as much as possible.

It can be seen from Section "Equal privacy budget allocation mechanism" that in differential privacy protection, there is a constraint relationship between information privacy disclosure and differential privacy budget parameter $\eta$. That is, for any object of attack capability, the formula for calculating the maximum mutual information privacy disclosure is:

23 $$ \max L\left( {\theta ,\eta } \right) = \log_{2} \frac{{\theta r^{\eta } }}{{\theta - 1 + r^{\eta } }} $$

**Figure 4:**
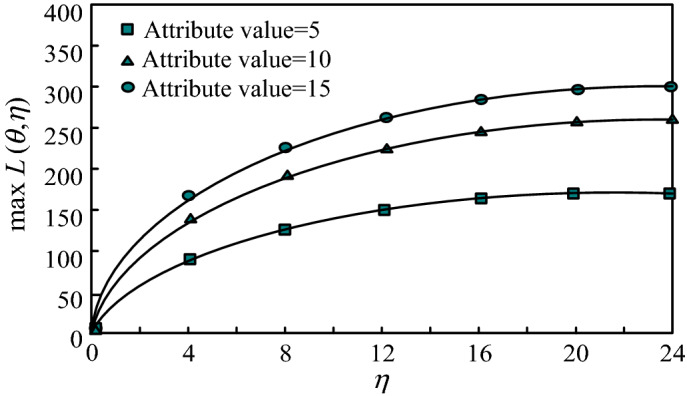
Maximum mutual information privacy leakage changing with privacy budget parameters.

In formula (23), the maximum privacy disclosure $\max L\left( {\theta ,\eta } \right)$ is a continuous function of privacy budget parameter $\eta$. When the privacy budget parameter is $\eta = 0$, the privacy protection level is the best, all lines in the differential privacy protection mechanism are the same, and there is no privacy disclosure in the differential privacy protection, that is, $\max L\left( {\theta ,\eta } \right) = 0$. On the contrary, when privacy budget parameter $\eta \to \infty$ is used, it is known that the privacy protection level is the worst, and the maximum privacy leakage $\max L\left( {\theta ,\eta } \right)$ converges to $\log_{2} v^{\eta }$. Set the data attribute values to 5,10,15, and the trend of maximum mutual information privacy disclosure $\max L\left( {\theta ,\eta } \right)$ in differential privacy protection with parameters $\theta$ and $\eta$ is shown in Fig. 4.

Figure 4 depicts the change of the maximum mutual information privacy leakage in differential privacy protection with privacy budget parameter $\eta$. The ordinate represents the maximum mutual information privacy leakage $\max L\left( {\theta ,\eta } \right)$, and the abscissa represents the value of privacy budget parameter $\eta$. It can be seen from Fig. 4 that the maximum mutual information privacy disclosure $\max L\left( {\theta ,\eta } \right)$ increases with the increase of privacy budget parameter values in differential privacy protection, indicating that the more values of attribute values are, the greater the maximum mutual information privacy disclosure is when the number of attributes in the dataset is the same. In addition, as the privacy budget tends to infinity, the maximum mutual information privacy disclosure $\max L\left( {\theta ,\eta } \right)$ converges to $\log_{2} v^{\eta }$. The above analysis shows that the algorithm in this paper can show the effectiveness of the maximum mutual information privacy disclosure. Analysis of the reasons for the advantageous results obtained in this article: Using TF-IDF method to extract network sensitive information text, collect word elements with high word frequency, and obtain more accurate text mining results for network sensitive information. Based on decision tree theory, the equal difference privacy budget allocation mechanism is improved to achieve equal difference privacy budget allocation, which can not only preserve the availability of original data, but also truly reproduce the structure of the original dataset. Random projection is used to perform dimensionality reduction and singular value decomposition on high-dimensional network graph data, and inverse operations are used to generate a matrix to be published, achieving effective protection of differential privacy for sensitive network information.

## Conclusion

At present, information differential privacy protection methods applied to various networks are mainly about small social networks. Although these privacy protection methods can resist background knowledge attacks to achieve the purpose of protecting social networks, with the advent of the big data era, the number of users increases and user attributes increase. These methods need to add a lot of noise, resulting in poor data availability. In order to reduce the risk of data privacy disclosure and improve the effect of information privacy protection, a differential privacy protection algorithm for network sensitive information based on singular value decomposition is proposed. The main innovations of the algorithm are as follows:

1. TF-IDF method is used to extract network sensitive information text, and the result of network sensitive information text mining is obtained, which provides a data basis for privacy protection.
2. Using singular value decomposition to disturb the confidential numerical attributes can not only meet the requirements of protecting sensitive data attributes, but also obtain accurate data analysis results.
3. By discarding some of its smaller singular values and corresponding spectral vectors, the data can be disturbed, which can not only retain the availability of the original data, but also truly represent the original data set structure.
4. The analysis of experimental results shows that this algorithm can show the effectiveness of the maximum mutual information privacy leakage, and the maximum data loss rate of this algorithm is only 5.8%, which fully verifies its application value.

## Data Availability

The datasets used and/or analysed during the current study available from the corresponding author on reasonable request.

## References

[1] Li K, Chen Y (2022). Fuzzy clustering-based financial data mining system analysis and design. Int. J. Found. Comput. Sci..

[2] Dudik R, Boruvka V, Riedl M, Holecek T (2021). Data mining and its impact on marketing communication - case: Heat-treated birch wood. Wood Res..

[3] Liu W, Peng YF, Tian Z, Sheng ZY, Li Y, She W (2021). A survey on medical information privacy protection based on blockchain. J. Zhengzhou Univ..

[4] Sun Z, Wang Y, Cai Z, Liu T, Tong X, Jiang N (2021). A two-stage privacy protection mechanism based on blockchain in mobile crowdsourcing. Int. J. Intell. Syst..

[5] Zou GH, Zou XY (2020). Research on differential privacy protection of sensitive information in swarm intelligence network. Comput. Simul..

[6] Zhang SX, Kang HY, Yan H (2019). Privacy preserving for social network relational data based on skyline computing. J. Comput. Appl..

[7] Fu YX, Qin YB, Shen GW (2019). Sensitive data privacy protection method based on transfer learning. J. Data Acquis. Process..

[8] Zwa C, Ss A, Xl B (2020). A dummy-based user privacy protection approach for text information retrieval—sciencedirect. Knowl.-Based Syst..

[9] Wu Z, Xuan S, Xie J (2022). How to ensure the confidentiality of electronic medical records on the cloud: A technical perspective. Comput. Biol. Med..

[10] Wu Z, Shen S, Li H (2021). A basic framework for privacy protection in personalized information retrieval: An effective framework for user privacy protection. J. Organ. End User Comput..

[11] Soltani O, Benabdelkader S (2022). Euclidean distance versus Manhattan distance for skin detection using the SFA database. Int. J. Biom..

[12] Barshandeh S, Dana R, Eskandarian P (2022). A learning automata-based hybrid MPA and JS algorithm for numerical optimization problems and its application on data clustering. Knowl.-Based Syst..

[13] Dou CH, Wei XY, Zhang JH, Hu L (2018). Fault feature enhancement of rolling bearings based on singular spectrum decomposition. J. Chin. Soc. Mech. Eng., Series C Trans. Chin. Soc. Mech. Eng..

[14] Mohammadiun S, Hu G, Gharahbagh AA, Mirshahi R, Li J, Hewage K, Sadiq R (2021). Optimization of integrated fuzzy decision tree and regression models for selection of oil spill response method in the Arctic. Knowled.-Based Syst..

[15] Kim Y, Park B, Kim SY (2022). A selective encryption/decryption method of sensitive music usage history information on theme, background and signal music blockchain network. J. Web. Eng..

[16] Wu Z, Li G, Shen S (2021). Constructing dummy query sequences to protect location privacy and query privacy in location-based services. World Wide Web.

[17] Wu Z, Shen S, Zhou H (2021). An effective approach for the protection of user commodity viewing privacy in E-commerce website. Knowled. Based Syst..

[18] Wu Z, Xie J, Shen S (2022). A confusion method for the protection of user topic privacy in Chinese keyword based book retrieval. ACM Trans. Asian Low-Res. Lang Inf. Process..

